# Morbidity and oncologic outcome after saphenous vein-sparing inguinal lymphadenectomy in melanoma patients

**DOI:** 10.1186/s12957-017-1164-x

**Published:** 2017-05-11

**Authors:** Johannes Baur, Katrin Mathe, Anja Gesierich, Gerhard Weyandt, Armin Wiegering, Christoph-Thomas Germer, Martin Gasser, Jörg O. W. Pelz

**Affiliations:** 1 Department of General, Visceral, Vascular and Pediatric Surgery, University Hospital Würzburg, Würzburg, Germany; 2 Department of Dermatology, Venerology and Allergology, University Hospital Würzburg, Würzburg, Germany

**Keywords:** Malignant melanoma, Inguinal lymph node dissection, Regional recurrence, V. saphena magna

## Abstract

**Background:**

Inguinal lymph node dissection (LND) is a surgical procedure with a high morbidity rate. Variations in surgical procedure, such as sparing of the saphenous vein, have been proposed to reduce surgical morbidity. While sparing of the saphenous vein has shown promising results in earlier studies, data for this procedure in melanoma patients are rare. In this retrospective study, we report 10-year findings on the effects of saphenous vein-sparing LND on surgical morbidity and oncologic outcomes in melanoma patients.

**Methods:**

A retrospective analysis of melanoma patients receiving inguinal LND in our facility between 2003 and 2013 was performed. Patients were divided into two groups: the saphenous vein resection group and the vein sparing group. Surgical morbidity, including wound infection, lymphatic fistula, severe bleeding, neurological complications, and chronic lymphedema, as well as regional recurrence-free survival were investigated.

**Results:**

A total of 106 patients were included in this study; of these, the saphenous vein was spared in 41 patients (38.7%). The rate of lymphatic fistula was 51.6 vs. 48.8%, wound infection occurred in 31.3 vs. 24.4%, and patients suffered from chronic lymphedema in 30.0 vs. 26.5% in V. saphena magna resection vs. sparing group. Differences observed, however, were not significant. No difference in regional recurrence-free survival between the two study groups was detected.

**Conclusions:**

The results of our retrospective analysis could not confirm the promising results reported in earlier studies. Thus, sparing of the saphenous vein appears to be optional.

## Background

In patients suffering from malignant melanoma, the standard treatment protocol is wide excision of the primary tumor followed by sentinel lymph node biopsy (SLNB) from the draining lymphatic basin if the tumor is ≥1 mm thick and complete inguinal lymph node dissection (LND) if metastatic involvement of the sentinel node is found. LND is also indicated if macro-metastases are detected during physical examination or in any cross-sectional study [1, 2]. The need for complete LND in case of positive SLNB remains controversial as this strategy does not seem to provide any survival benefit compared with careful observation and performing delayed LND when macro-metastases become evident at follow-up [3, 4]. Nevertheless, inguinal LND remains part of therapy for patients with melanomas of the lower limb and trunk when evident macro-metastases are detected during pretreatment staging or during follow-up.

Inguinal LND is a surgical procedure with a high morbidity rate and wound complication rates of up to 71% [5, 6]. Many variations in surgical technique, such as sartorius transposition or saphenous vein-sparing LND, have been proposed to reduce procedural morbidity [7, 8]. A randomized controlled trial revealed that sartorius transposition presents no advantages over conventional inguinal LND. By contrast, three retrospective studies on saphenous vein-sparing inguinal LND showed that the procedure yields lower rates of lymphedema [9–11]. In these studies, surgery was performed in patients with vulvar cancer.

Studies on variations in LND procedures in melanoma patients are rare. A retrospective study showing no difference in postoperative wound breakdown has been reported, and this study provides no further information related to other morbidities or oncologic outcomes [12]. No persistent lymphedema formation 18 months after saphenous vein-sparing inguinal LND was reported by the only prospective single-arm study available thus far [8]. Despite its obvious strengths, however, this study was missing a control arm and used only a small study population, thereby weakening the impacts of its results. Furthermore, oncologic outcomes in this work were investigated only rudimentarily because the mean follow-up period was only 14.8 months.

In this retrospective study, we report 10-year findings on the effects of saphenous vein-sparing LND on surgical morbidity and oncologic outcomes in melanoma patients.

## Methods

### Patients

Patients treated at our facility for malignant melanoma between 2003 and 2013 were investigated retrospectively and included in the study if (1) they suffered from melanoma of the trunk or the legs; (2) they received regional inguinal LND; (3) they received LND with curative intention; (4) vena saphena magna was mentioned in their operative report and the vein has not been resected in any previous surgical procedure, and (5) no in-transit or distant metastases was detected at the time LND was performed. Indications for LND were either positive sentinel node biopsy (SNB) or inguinal lymph nodes, suspicious for macro-metastases during physical exam and/or any cross-sectional study (usually sonography or computed tomography), in the course of pretreatment staging or follow-up. Basic patient and tumor characteristics, as well as surgical morbidity (lymphatic fistula, wound infection, severe bleeding, neurological complication, need for surgical revision, chronic lymphedema) and regional recurrence-free survival, were investigated.

### Surgical procedure

Standard inguinal LND was performed as described elsewhere [13]. In brief, en bloc removal of all inguinal lymphatic tissues contained within the femoral triangle caudally to the inguinal ligament was performed. However, according to the German melanoma guidelines, lymph nodes dorsally the fascia lata (deep inguinal dissection) are not dissected in our institution. The decision to spare the vena saphena magna was left to the performing surgeon.

### Postoperative management

All of the patients in our facility received one or two surgical site drains and compression stockings after inguinal LND. Drains were removed only when they produced less than 10 ml of lymphatic fluid per day. Patients were discharged from the hospital when wound stability with non-irritated wound condition without purulent discharge was achieved and they were able to manage their surgical site drains, in case the drains had to remain longer. Patients were routinely controlled following their hospital stay until wound stability was reached, and all drains were removed. Wound infections were treated with either antibiotics or surgical revision. As this study was carried out over a span of 10 consecutive years, the possibility that postoperative standards may have changed throughout the investigation period was considered.

### Definitions of surgical complications

Lymphatic fistulas are defined as persistent secretion of lymphatic fluid for 21 days or more after LND. Wound infection was assumed if typical symptoms (e.g., redness, purulent discharge, abscess formation) were detected. Wound swabs were not routinely performed during postoperative care. Neurologic complication was assumed if symptoms such as postoperative paresthesia or motoric deficits were detected. Severe bleeding was defined as bleeding requiring surgical revision to staunch.

The presence of chronic lymphedema was investigated 6 and 12 months postoperatively. Chronic lymphedema was assumed when patients showed typical signs during physical examination like an obvious increase in the circumference of the operated limb or a momentary indentation after pressing the pretibial tissue with the finger tips. Chronic lymphedema was also assumed when patients reported complaints such as “swollen leg” or “persistent need (once a week or more often) for physical lymphatic drainage.”

### Postoperative oncologic treatment and local recurrence

All cases of malignant melanoma in our facility were discussed in an interdisciplinary tumor board. The need for adjuvant treatment was determined according to German melanoma guidelines [1, 2]. In patients in which regional recurrence occurred, only the systemic therapies before this time point were recorded. In patients which did not experience regional recurrence, all systemic therapies after LND were recorded.

Patients underwent follow-up examinations periodically according to the recommendations provided in German melanoma guidelines. Follow-ups included physical examination of the whole integument and ultrasound of the regional lymph node basin every 3–6 months during the first 5 years after primary tumor excision. Additional cross-sectional studies were performed in advanced clinical stages every 6 months during the first 3 years after primary tumor excision. The time of regional recurrence was determined if masses with potential malignancy were detected in the region of LND in any postoperative cross-sectional study.

### Statistical analysis

Statistical analysis between the two study groups was performed using the *χ* ^2^ test to determine categorical endpoints. A *t* test was used to analyze continuous endpoints, and a log-rank test was used to analyze survival endpoints. Differences were considered significant when *p* values <0.05.

## Results

### Baseline patient and tumor characteristics

Baseline patient and tumor characteristics are shown in Table 1. A total of 106 patients with an average age of 58.3 years were included in this study; of these, 46.2% were male. V. saphena sparing (SaphSpar) inguinal LND was performed in 38.7% of the patients. No statistically significant difference in sex, age, body mass index (BMI), nicotine abuse, diabetes mellitus, or arterial hypertonia was observed between groups. No difference in operative time and mean hospital stay was found, and no difference in distribution of tumor localization, type of melanoma, lymph node status, and primary tumor thickness could be detected. Similar mean numbers of lymph nodes were harvested from both groups, although the V. saphena magna resecting (SaphRes) group showed a slightly higher numbers of resected lymph nodes than the SaphSpar group (9.0 vs. 7.9 lymph nodes, *p* = 0.2318). The mean follow-up time for all patients was 38.6 months. Follow-up was statistically significant longer in the SaphSpar group than in SaphRes group (53.1 vs. 29.5 months, *p* = 0.0006).

**Table 1:** Baseline clinical and pathologic characteristics of patients

		Overall	(%)	SaphRes	(%)	SaphSpar	(%)	*p* value
Patients								
No. of patients		106		65	(61.3)	41	(38.7)	
Male sex		49	(46.2)	30	(46.2)	19	(46.3)	0.9849
Age	[years]	58.3		59.7		56.2		0.2239
BMI	[kg/m^2^]	26.9		26.8		27.2		0.4902
Risk factors								
Nicotine abuse		24	(22.6)	12	(18.5)	12	(29.3)	0.1954
Diabetes mellitus		9	(8.5)	5	(7.7)	4	(9.8)	0.7105
Arterial hypertonia		49	(46.2)	30	(46.2)	19	(46.3)	0.9849
Surgical procedure								
Mean duration	[min]	87.0		83.9		91.8		0.2955
Mean hospital stay	[days]	12.0		11.6		12.6		0.6372
Mean no. resected LN		8.6		9.0		7.9		0.2318
Primary tumor characteristics								
Location								
Located at limb		81	(76.4)	50	(76.9)	31	(75.6)	
Located at trunk		18	(17.0)	10	(15.4)	8	(19.5)	0.7560^a^
Unknown		7	(6.6)	5	(7.7)	2	(4.9)	
Melanoma subtype								
SSM		45	(42.5)	25	(38.5)	20	(48.8)	
NM		16	(15.1)	11	(16.9)	5	(12.2)	0.7303^b^
Other		35	(33.0)	23	(35.4)	12	(29.3)	
Unknown		10	(9.4)	6	(9.2)	4	(9.8)	
T stage								
pT1		9	(8.5)	5	(7.7)	4	(9.8)	
pT2		26	(24.5)	16	(24.6)	10	(24.4)	
pT3		24	(22.6)	14	(21.5)	10	(24.4)	0.9763^c^
pT4		21	(19.8)	14	(21.5)	7	(17.1)	
Unknown		26	(24.5)	16	(24.6)	10	(24.4)	
Ulcerated		44	(41.9)	29	(44.6)	15	(37.5)	0.4730
Mean thickness	[mm]	3.5		4.5		3.1		0.3542
Lymph node status								
Positive SLN		53	(50.0)	30	(46.2)	23	(56.1)	0.3187
Positive non-sentinel LN		51	(48.1)	31	(47.7)	20	(48.8)	0.9130
Positive LN status^d^		89	(84.0)	53	(81.5)	36	(87.8)	0.3919
Follow-up								
Mean	[months]	38.6		29.5		53.1		0.0006

^a^Location: "Located at limb" vs. "Located at trunk" vs. "Unknown"

^b^Melanoma subtype: "SSM" vs. "NM" vs. "Other" vs. "Unknown"

^c^T-Stage: "pT1" vs. "pT2" vs. "pT3" vs. "pT4" vs. "Unkown"

^d^Positive SLN or positive non-sentinel LN

*no.* number, *SaphRes* V. saphena magna resection, *SaphSpar* V. saphena magna sparing, *BMI* body mass index, *SSM* superficial spreading melanoma, *NM* nodular melanoma, *SLN* sentinel lymph node, *LN* lymph node

### Surgical morbidity after LND

Rates of surgical morbidity after LND are shown in Table 2. Lymphatic fistulas occurred in 50.5% and wound infections in 28.6% of cases in overall study collective. Complications like severe bleeding or neurological complications were much rarer (5.7 and 1.9% respectively).

**Table 2:** Surgical morbidity after LND. Lymphatic fistulas are defined as persistent secretion of lymphatic fluid for 21 days or more after LND. Wound infection was assumed if any typical symptoms (e. g., redness, purulent discharge, abscess formation) were detected. Neurological complications were defined if symptoms like postoperative paraesthesia or loss of motor function was detected. Severe bleeding was defined as bleeding that needs to be surgically revised

		Overall	SaphRes	SaphSpar	*p* value
Lympatic fistula	[%]	50.5	51.6	48.8	0.7809
Wound infection	[%]	28.6	31.3	24.4	0.4478
Severe bleeding	[%]	5.7	7.7	2.4	0.2543
Neurological complications	[%]	1.9	1.5	2.4	0.7400
Need for surgical revision	[%]	18.9	20.0	17.1	0.7076
Lymphedema 6 months after LND^a^	[%]	28.6	30.0	26.5	0.7252
Lymphedema 12 months after LND^b^	[%]	20.9	22.9	18.8	0.6796

^a^Only patients with a complete 6-month follow-up after LND were considered. Data available in 84 cases (50 SaphRes, 34 SaphSpar)

^b^Only patients with a complete 12-month follow-up after LND were considered. Data available in 67 cases (35 SaphRes, 32 SaphSpar)

Surgical revision within the first 30 days after LND was necessary in 18.9% of cases. In 6 patients, re-operation was required due to severe bleeding and in 14 cases infectious wound complication including infected lymphoceles was the reason for surgical revision.

No statistically significant difference in terms of occurrence of early postoperative complications was observed between the SaphRes and SaphSpar groups. Chronic lymphedema postoperatively represented a frequent complication. Data regarding the occurrence of chronic lymphedema 6 months after LND were obtained from 84 patients (50 patients in the SaphRes group, 34 patients in the SaphSpar group). Overall, 28.6% of these patients either showed lymphedema during physical examination or reported lymphedema-associated discomforts (e.g., swollen leg) during anamnesis. The rate of chronic lymphedema 12 months after LND was slightly lower and added up to 20.9% in the overall study population. Here, data was available in 67 cases (35 patients in the SaphRes group, 32 patients in the SaphSpar group). With regard to chronic lymphedema, there were also no statistically significant differences between the two investigated study groups. After 6 months, the rate of chronic lymphedema was 30.0% in the SaphRes vs. 26.5% in the SaphSpar group (*p* = 0.7252) and 22.9 vs. 18.9% (*p* = 0.6796) after 12 months respectively.

The influence of patient-related risk factors on surgical morbidity was investigated, and results are shown in Table 3. Nicotine abuse, diabetes mellitus, and arterial hypertonia did not show any statistically significant influence on surgical morbidity. However, patients with arterial hypertonia tended to have a higher risk for severe postoperative bleeding. A BMI >30 kg/m^2^ was also associated with an increased risk of surgical morbidity. In particular, 80% of the patients with BMI >30 kg/m^2^ suffered from the early postoperative complications (lymphatic fistula, wound infection, severe bleeding, neurological complications) compared with 52.5% of the patients with a BMI ≤30 kg/m^2^ (*p* = 0.0108). Subgroup analysis of patients with BMI >30 kg/m^2^, however, revealed that SaphSpar inguinal LND did not necessarily reduce surgical morbidity in these patients (data not shown).

**Table 3:** Influence of patient-related risk factors on surgial morbidity. Lymphatic fistulas are defined as persistent secretion of lymphatic fluid for 21 days or more after LND. Wound infection was assumed if any typical symptoms (e. g., redness, purulent discharge, abscess formation) were detected. Neurological complications were defined if symptoms like postoperative paraesthesia, or loss of motor function were detected. Severe bleeding was defined as bleeding that needs to be surgically revised

		Nicotine abuse			Diabetes mellitus			Arterial hypertonia			BMI >30		
		Yes	No	*p* value	Yes	No	*p* value	Yes	No	*p* value	Yes	No	*p* value
Lympatic fistula	[%]	37.5	54.3	0.1477	66.7	49.0	0.3096	55.1	46.4	0.3752	65.4	45.6	0.0796
Wound infection	[%]	25.0	29.6	0.6592	22.2	29.2	0.6592	36.7	21.4	0.0833	42.3	24.1	0.0739
Severe bleeding	[%]	4.2	6.1	0.7188	11.1	5.2	0.4595	10.2	1.8	0.0605	57.7	5.0	0.6058
Neurological complications	[%]	0.0	2.4	0.4399	11.1	1.0	0.0335	2.0	1.8	0.9139	3.8	1.3	0.3980
Lymphedema^a^	[%]	28.6	27.7	0.9378	28.6	27.8	0.9674	27.0	28.6	0.8744	36.4	25.0	0.3053

^a^Only patients with a complete 6-month follow-up after LND were considered

### Regional recurrence-free survival

**Fig. 1:**
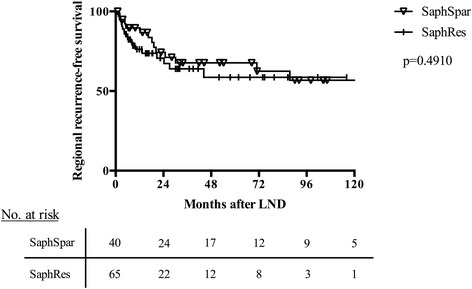
Regional recurrence-free survival of the patients included in this study. No significant difference between the SaphRes and SaphSpar groups was observed

**Fig. 2:**
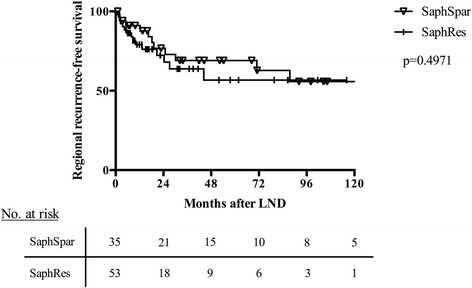
Regional recurrence-free survival of patients with positive lymph node status (i.e., positive sentinel and non-sentinel lymph nodes). No significant difference between the SaphRes and SaphSpar groups was observed

The overall local recurrence-free survival of both groups is shown in Fig. 1. No statistically significant difference between the SaphRes and SaphSpar groups was found (*p* = 0.3368). Subgroup analysis for patients with positive lymph node status (i.e., positive sentinel lymph node and/or positive lymph nodes in dissected tissues) was performed, and no difference between the two study groups was detected (Fig. 2). Systemic therapies the patients received after LND are shown in Table 4. There were no differences between the two study groups regarding the applied systemic therapies.

**Table 4:** Systemic therapies after LND. Additional systemic therapies are shown. Only therapies after LND and before occurrence of regional recurrence has been recorded

		Overall	SaphRes	SaphSpar	*p* value
Interferone	[%]	17.0	21.0	10.5	0.1773
Any chemotherapy	[%]	20.0	17.7	23.7	0.4709
Dacarbazine	[%]	17.0	14.5	21.1	0.3983
Paclitaxel	[%]	6.0	6.5	5.3	0.8081
Cisplatin	[%]	4.0	3.2	5.3	0.6138
Vemurafenib	[%]	1.0	0.0	2.6	0.1992
Sorafenib	[%]	1.0	1.6	0.0	0.4314
Ipilimumab	[%]	7.0	6.5	7.9	0.7837
Vaccination	[%]	6.0	6.5	5.3	0.8081

## Discussion

Inguinal LND is a surgical procedure with high morbidity rates. The most frequent complications are wound infection and formation of lymphatic fistulas or seromas [12]. These complications mostly result in long hospital stays, consecutive long-term out-hospital treatment, and restriction of quality of life. The literature reports wound infection rates of about 8–55% of all LND cases [14–16]. Some studies even report wound complications, including wound infections, delayed healing, and wound breakdown, occurring in over 70% of all cases [6, 12]. Lymphatic fistulas and seroma formation have been reported in 12–30% of all LND cases [8, 9, 15, 16]. This inconsistency in the so far published data results from the mostly retrospective design of the studies and missing common accepted definitions of these complications. Nevertheless, the high rate of surgical complications observed after inguinal LND has led to several changes in the procedural technique in order to reduce high complication rates. For example, transposition of the sartorius muscle was applied for defect closure in one prospective [7] and one retrospective study [17]. Both of these studies, however, did not show a reduction in seroma formation.

Preservation of the V. saphena magna represents another adjustment to the surgical procedure of inguinal LND; this technique has mainly been investigated in retrospective studies. Three retrospective studies showing a reduction in the rate of chronic lymphedema after V. saphena magna preservation have been reported. In these studies, chronic lymphedema rates of 38.7, 48.3, and 70% were reported among patients with resected V. saphena magna compared with significantly reduced rates of 11, 32, and 25%, respectively, among patients with spared veins [9–11]. Only two studies examining saphena vein-sparing inguinal LND in melanoma patients have been published. One retrospective study showed that vein sparing yielded no advantages over vein resection in terms of occurrence of complications [12].

The only prospective study available regarding this topic reported a chronic lymphedema occurrence rate of 28.5% at 6 months postoperatively; furthermore, no patient suffered from lymphedema 2 years after the operation [9]. In 2011, Abbas et al. performed a systemic review and meta-analysis to investigate the effects of preserving the saphenous vein; here, the superiority of saphenous vein-sparing inguinal LND over conventional inguinal LND in terms of occurrence of wound breakdown and lymphedema was demonstrated [18].

In the present retrospective study, we present our 10-year findings on V. saphena magna-sparing inguinal LND in melanoma patients. A total of 106 melanoma patients receiving this surgical procedure at our facility between 2003 and 2013 were included in this study, and the impact of vein sparing on surgical morbidity and local recurrence-free survival was investigated. To the best of our knowledge, this work represents the largest study published thus far investigating this technique in melanoma patients. Sparing of the saphenous vein did not appear to promote reductions in the occurrence of early postoperative complications, such as lymphatic fistula or wound infection, or chronic lymphedema 6 and 12 months postoperatively. This result contrasts findings reported in other studies, and the root of this difference remains unclear. We believe that the retrospective design of the most studies and the non-standardized definitions of the complications may have contributed to the differences observed.

The effect of preserving or resecting the saphenous vein on oncologic outcome in melanoma patients has thus far only been investigated rudimentarily. Only one prospective single-arm study has yielded any information on this topic; in this study, the mean follow-up period was 14.8 months and one patient developed pulmonary metastases 6 months postoperatively. Despite its obvious strengths, however, a missing control arm, low number of study participants, and relatively short mean follow-up period diminish the reliability of this work in terms of describing oncologic outcomes.

The present study provides valid information regarding the effects of saphenous vein-sparing inguinal LND on oncologic outcome parameters, such as regional recurrence-free survival, in melanoma patients for the first time. The mean follow-up period in this work was 38.6 months. Patients in the SaphSpar group reported a significantly higher mean follow-up period of 53.1 months compared with the 29.5 months of the SaphRes group. This difference may be attributed to the long time span over which this study was performed and consecutive changes in surgeons performing LND.

The number of lymph nodes during excised complete LND is known to be an independent predictor of overall survival [19, 20] in melanoma patients. Although not statistically significant, the mean number of excised lymph nodes was slightly higher in SaphRes patients than in SaphSpar patients (9.0 vs. 7.9). This difference, however, did not affect the overall regional recurrence-free survival of both groups.

About 16% of the patients in this study revealed a negative lymph node status (i.e., negative sentinel and non-sentinel lymph nodes). These patients received inguinal LND because of inguinal lymph nodes, suspicious for macro-metastases during physical exam and/or any cross-sectional study (usually sonography or computed tomography), particularly at the pretreatment staging or follow-up. Pathological reports, however, revealed that the regional lymphatic nodes were not involved in the original melanoma. As patients with a positive lymph node status (i.e., positive SLNB or positive non-sentinel lymph nodes) may benefit from more radical operations, subgroup analysis was performed; no significant differences in regional recurrence-free survival was observed between the two study groups (Fig. 2).

Limitations of the study are its retrospective design and the relative low number of inguinal LND procedures performed in a quite long time span of 10 years. However, as some patients could not be included in this study according to our inclusion criteria, the procedures investigated in this study do not reflect all inguinal LNDs performed in our institution during this interval. Also, the number of inguinal LNDs performed in our institution per year is comparable to other certified melanoma centers in Germany and as such, only two experienced senior surgeons perform or supervise inguinal LND in our facility to ensure a certain procedural quality. This leads to similar results in quality markers like rate of wound and overall complications [21] as well as duration of the hospital stay [5] in our study collective compared to other studies published so far regarding inguinal LND. So, the risk for bias resulting from the number of LNDs performed in our institution is assumed to be low.

## Conclusions

In conclusion, our results indicate that resection or sparing of the saphenous vein is facultative. In this study, saphenous vein-sparing inguinal LND did not improve surgical morbidities and resection did not improve oncologic outcomes.

## Abbreviations

BMI: Body mass index
LN: Lymph nodes
LND: Lymphadenectomy
NM: Nodular melanoma
SaphRes: V. saphena magna resecting
SaphSpar: V. saphena magna sparing
SLNB: Sentinel lymph node biopsy
SSM: Superficial spreading melanoma
